# Restoration of immune surface molecules in Kaposi sarcoma-associated herpes virus infected cells by lenalidomide and pomalidomide

**DOI:** 10.18632/oncotarget.17960

**Published:** 2017-05-17

**Authors:** David A. Davis, Suraj Mishra, Holda A. Anagho, Ashley I. Aisabor, Prabha Shrestha, Victoria Wang, Yuki Takamatsu, Kenji Maeda, Hiroaki Mitsuya, Jerome B. Zeldis, Robert Yarchoan

**Affiliations:** ^1^ HIV and AIDS Malignancy Branch, Center for Cancer Research, National Cancer Institute, Bethesda, MD, USA; ^2^ Celgene Corp., Summit, NJ, USA

**Keywords:** Kaposi sarcoma-associated herpesvirus, pomalidomide, lenalidomide, major histocompatibility class I, ICAM-1

## Abstract

Kaposi sarcoma-associated herpesvirus (KSHV) is the cause of several tumors, including Kaposi sarcoma and primary effusion lymphoma (PEL). Most viruses have evolved means of escaping immune recognition. KSHV downregulates MHC-I expression during lytic infection, and expression of ICAM-1 and B7-2 (CD86) during latent infection, allowing evasion of T cell and natural killer immunity respectively. These effects are largely mediated by two KSHV-encoded proteins, K3 and K5. We show here that lenalidomide (Len) and pomalidomide (Pom) prevent down-regulation of MHC-I during lytic activation, and restore ICAM-1 and B7-2 surface expression in latently infected PEL cells. Importantly, these changes occurred at clinically achievable concentrations and prior to any cytotoxic effects. Exploration of the mechanism revealed that Pom blocked lytic down-regulation of MHC-I induced by transfection with K3 but not K5. Although Pom alone did not significantly increase HLA mRNA expression in PEL cells, it did blunt the butyrate-induced decrease in MHC-I mRNA expression and decreased the upregulation of K3 mRNA in lytic cells. Virus-induced tumors express foreign antigens, but immunotherapy can be thwarted by viral strategies to evade immune recognition. The effects of Pom and Len described here can prevent these strategies and support the use of these drugs to treat KSHV-induced tumors.

## INTRODUCTION

Kaposi's sarcoma-associated herpesvirus (KSHV), also known as human herpesvirus 8 (HHV-8), is an oncogenic virus that is the causative agent of two tumors, Kaposi's sarcoma (KS) and primary effusion lymphoma (PEL); a B cell hyperproliferative disease, multicentric Castleman's disease; and an inflammatory cytokine syndrome, KSHV inflammatory cytokine syndrome [1–4]. These conditions predominantly arise in HIV-infected patients. KSHV establishes a latent infection in target cells including B-cells and endothelial cells. A limited number of viral proteins expressed in latency maintain the viral genome, enhance target cell survival, and function to curtail anti-viral strategies of the host. Several viral proteins help prevent immune recognition in latency. For example, latency-associated nuclear antigen (LANA) interferes with MHC-I expression and antigen presentation [5]. Latent cells can undergo lytic activation resulting in expression of all KSHV genes and production of progeny virus. KSHV has evolved additional mechanisms to avoid immune recognition during lytic activation when more viral genes are expressed. K3 and K5 are two virus-encoded E3 ubiquitin ligases that are upregulated upon lytic activation and down-regulate MHC-I through ubiquitination and degradation [6–9]. By this mechanism K3 and K5 can render cells invisible to cytotoxic T-cells. In addition, K5, which is expressed very early upon infection and at a low level during latency [10], can down-regulate expression of ICAM-1 (CD54) and B7-2 (CD86) [11], which are ligands for natural killer (NK) cell mediated cytotoxicity. Inhibition of their expression can block killing by NK cells, and reestablishing their expression restores killing [11]. Other KSHV-encoded proteins also contribute to down-regulation of surface immune recognition molecules. For example, KSHV replication and transcription activator (RTA) down-regulates MHC class II expression [12], and vIRF-1 is able to prevent viral FLICE inhibitory protein (vFLIP)-induced expression of MHC-I mRNA in endothelial cells [13]. These lytic proteins are also transiently produced during *de novo* KSHV infection and contribute to establishment of latency by avoiding immune recognition [10].

Thalidomide (Tha) is an effective treatment for multiple myeloma (MM), and two analogs of Tha, lenalidomide (Len) and pomalidomide (Pom), have more recently been approved for MM and are more effective than Tha; Len is also approved for mantle cell lymphoma and myelodysplastic syndromes [14, 15]. The principal target of these drugs is cereblon, a component of certain cullin-4 (CUL4) E3 ubiquitin ligase complexes that provides substrate specificity [16–20]. Many anti-tumor effects of these drugs are related to an increase in degradation of transcription factors Aiolos and Ikaros (encoded by IKZF-3 and IKZF-1 respectively); this in turn can lead to down-regulation of c-Myc and interferon regulatory factor 4 (IRF4) in MM cells, and also to immunomodulation and effector T cell co-stimulation [17, 21]. Len and Pom also inhibit NF-κB in diffuse large B cell lymphoma (DLBCL) and MM, and this, along with effects on IRF4, is associated with inhibition of cell growth and cellular toxicity [22, 23]. These drugs have been reported to be cytotoxic to PEL cells and to display synergistic toxicity with BRD4 inhibitors [24]. Our group showed Tha has some clinical activity against KS, and it has more recently been reported that Len and Pom have substantial clinical anti-KS activity at doses that are well-tolerated [25, 26]. Also, there is a report that Len was effective in a patient with PEL [27].

We explored the possibility that one of the reasons for the activity of Tha, Len, and Pom against KSHV-induced tumors might be that they prevented KSHV-induced downregulation of surface immune recognition molecules by the activity of K3 or K5, or enhanced immunologic recognition by other mechanisms [28]. In this study, we found that these immunomodulatory agents prevent down-regulation of MHC-I surface expression during lytic activation in KSHV infected cells and restore ICAM-1 and B7-2 expression in latent cells. Pom also restored MHC-I expression in K3 transfected cells. Interestingly, Pom prevented a decrease in MHC-I mRNA transcription during lytic activation, which could account at least in part for its effects on MHC-I expression. Pom also decreased K3 expression in lytically activated cells, but not latently infected cells. It is unclear if Pom also affects surface immune molecules by other mechanisms. This novel finding suggests these drugs not only inhibit PEL growth but can also disrupt viral immune evasion mechanisms, thus providing a rationale for their use in the treatment of KSHV-induced tumors.

## RESULTS

### Len and Pom inhibit KSHV-induced lytic down-regulation of MHC I expression

**Figure 1:**
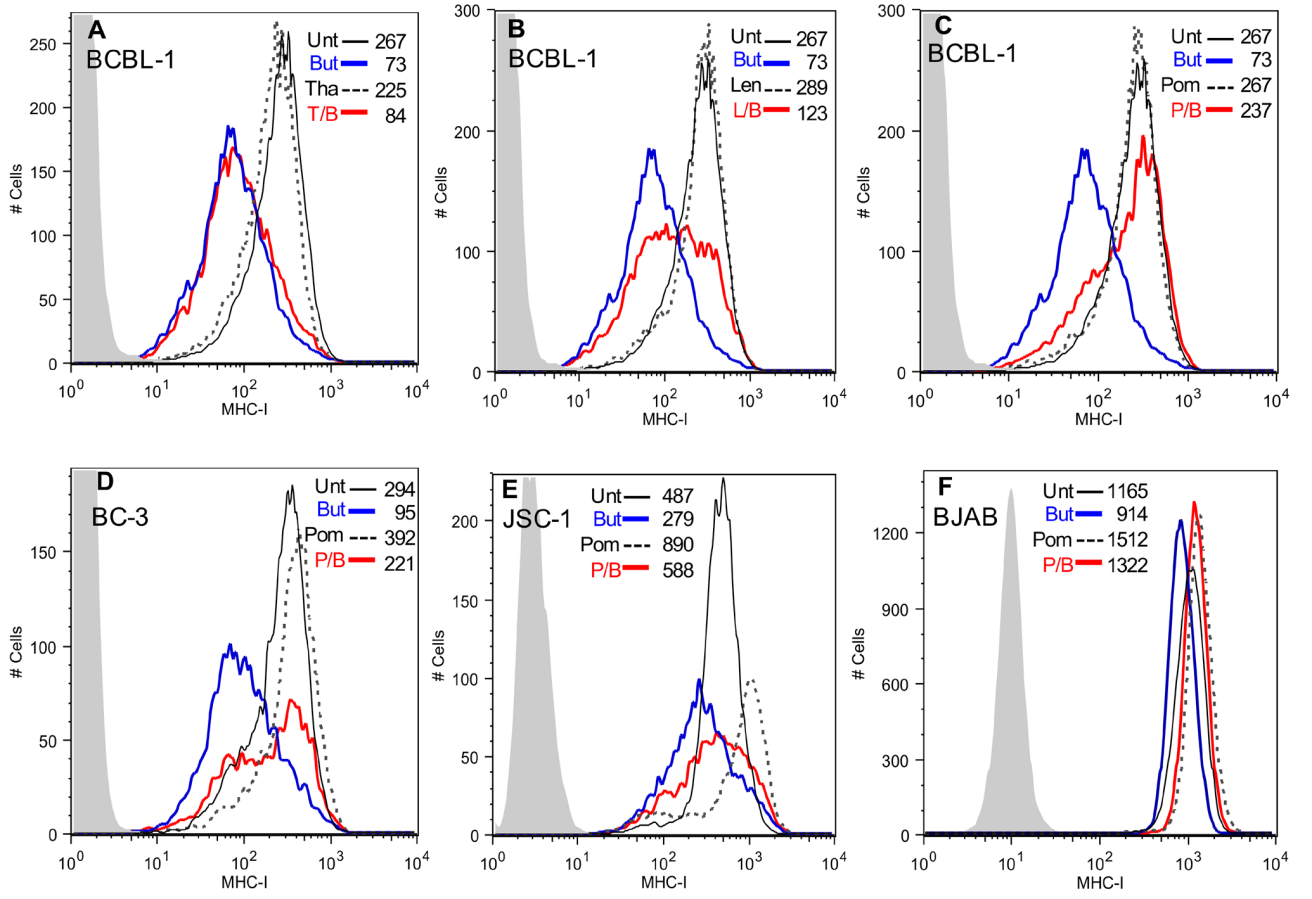
Effect of Tha, Len and Pom on the lytic-induced down-regulation of MHC-I in KSHV infected cells Cells (300,000 cells/ml in 10 ml) were pretreated for 24 h with Tha, Len or Pom or DMSO vehicle control. Cells were then treated with sodium butyrate (0.3 mM) for another 24 h and then analyzed by flow cytometry for surface protein expression. Cells were washed with cold FACS buffer (10% FBS, 0.1% sodium azide in PBS) and incubated with conjugated monoclonal antibodies (2 μL) for 30 min at 4°C. After washing twice, MHC-I expression was determined by FACS. **A**. BCBL-1 cells pretreated for 24 h with DMSO vehicle control (Unt) or 10 μM Tha, **B**. 10 μM Len or **C**. 10 μM Pom, followed by treatment with PBS or 0.3 mM butyrate (But). T/B, L/B, and P/B indicate Tha, Len, or Pom pretreatment followed by butyrate 24 hrs later. MHC-I expression by FACS in BC-3 cells **D**., JSC-1 cells **E**., or BJAB cells **F**. pre-treated with DMSO vehicle control (Unt) or 10 μM Pom followed by treatment with PBS or 0.3 mM butyrate (But) 24 h before FACS analysis for MHC I expression. Results shown are representative of 3 separate experiments in A-E and 2 experiments in F. The isotype control is shown in grey shading in each Figure. Control cells (solid black line, Pom, Len or Tha treated cells (dashed black line, butyrate treated cells (blue line) and drug then butyrate treated (T/B, L/B, and P/B) (red line). The median fluorescent value is indicated within each Figure.

We investigated the effects of the immunomodulatory drugs Tha, Len, and Pom, on KSHV-induced down-regulation of MHC-I. As expected [29], PEL cells induced to lytic activation with butyrate exhibited a substantial down-regulation of MHC-I expression (73% decrease in median fluorescence compared to control) (Figure 1A, 1D, and 1E, compare blue line to solid black line). Pretreatment of BCBL-1 cells with 10 μM Tha had essentially no effect on down-regulation of MHC-I expression by butyrate (red line) (Figure 1A). However, 10 μM Len (Figure 1B) or Pom (Figure 1C) substantially prevented butyrate-induced down-regulation of MHC-I (Figure 1B, 1C, compare blue line to red line). By contrast, the drugs alone had little effect on the basal MHC-I expression in BCBL-1 cells (Figure 1A, compare dashed to solid black lines). Pom was most effective on a molar basis at preventing lytic downregulation of MHC-I and inhibited 85% of the decrease in MHC-I expression induced by butyrate (Figure 1C) while Len inhibited 26% of MHC-I expression induced by butyrate (Figure 1B). These effects by Len and Pom were also observed at clinically achievable concentrations (Cmax of Len 2 μM and for Pom 0.3 μM [30, 31]), with 30% and 63% inhibition of the decrease in MHC-I expression, respectively (Supplementary Figure S1A & B).

**Figure 2:**
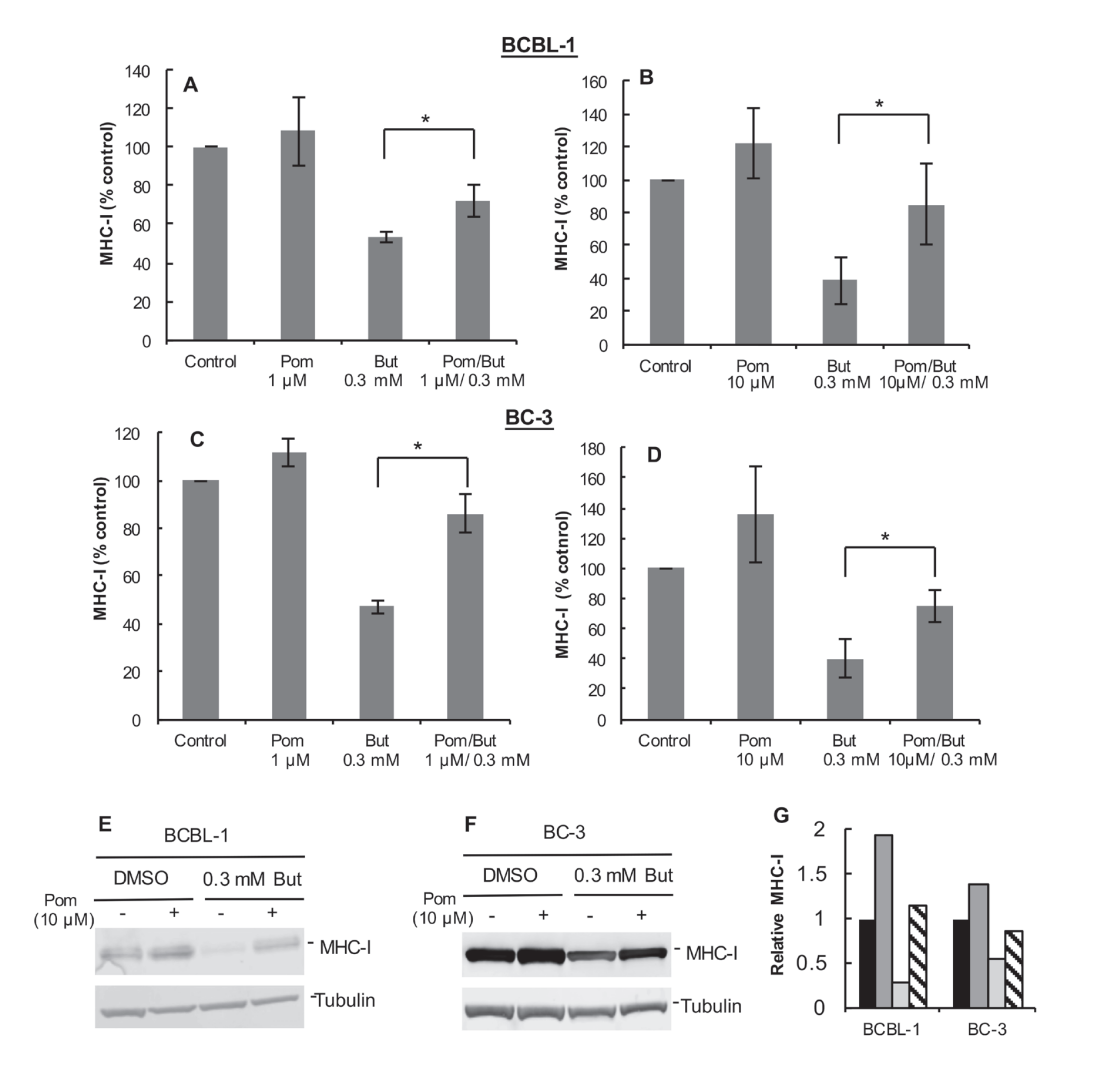
Pretreatment with Pom at 1 μM or 10 μM prevents the KSHV lytic-induced down-regulation of MHC-I in BCBL-1 and BC-3 cells MHC-I expression as determined by FACS in BCBL-1 cells pretreated with 1 μM Pom **A**. or 10 μM Pom **B**. or in BC-3 cells pretreated with 1 μM Pom **C**. or 10 μM Pom **D**.. Cells were pretreated with DMSO vehicle control (Control), 1 μM Pom, or 10 μM Pom. After 24 h cells were treated with 0.3 mM butyrate to induce lytic replication and then analyzed for MHC-I expression by FACS. The average median fluorescent value for each control was set at 100% and the treatment values are percent of the control. Values plotted are the average +/- standard deviation from four experiments for each cell line. Statistics were done using Student's two tailed paired *t*-test, *P < 0.05. **E**. Immunoblot analysis of MHC-I expression in cytoplasmic extracts from BCBL-1 cells pretreated for 24 h with 10 μM Pom followed by 0.3 mM butyrate treatment for 24 h or **F**. BC-3 cells. Tubulin was used as a loading control. **G**. Relative MHC-I protein expression from immunoblots of BCBL-1 cells and BC-3 cells normalized to tubulin control using the Licor analysis system. Legend: Black bars, DMSO vehicle control; Dark grey bars; 10 μM Pom; Light grey bars, 0.3 mM But; Diagonal hatched bars, 10 μM Pom/0.3 mM But.

We also investigated the ability of Pom to affect MHC-I expression in BC-3 and JSC-1 PEL cells and in uninfected BJAB B-cells. Interestingly, in these cell lines 10 μM Pom treatment alone led to discernable increases in MHC-I expression over control levels in the absence of sodium butyrate (MHC-I expression increased 33%, 82%, and 30% over control for BC-3, JSC-1, and BJAB, respectively) (Figure 1D-1F). Pom pretreatment also prevented the MHC-I down-regulation by butyrate in BC-3 and JSC-1 but appeared somewhat less effective than in BCBL-1 cells (Figure 1D and 1E). For example, butyrate treatment caused a 68% decrease in MHC-I expression in BC-3 cells and pretreatment with 10 μM Pom inhibited 63% of this decrease (Figure 1D, compare blue line to red line). In the uninfected B-cell line, BJAB, butyrate slightly decreased MHC-I expression (from median fluorescence of 1165 to 914) and this was prevented with Pom (Figure 1F). Further studies indicated that Pom at 1 μM also prevented down-regulation of MHC-I in PEL cells from butyrate treatment and that this effect was statistically significant at both 1μM and 10μM (Figure 2A-2D). Although there was a trend towards increased baseline MHC-I expression after exposure to 10 μM Pom alone in both cell lines, this change was less pronounced than the effects on butyrate-treated cells (Figure 2B and 2D).

It has previously been reported that Len and Pom can reduce the growth of PEL cells [24]. It is important to point out, however, that at the time that the MHC-I expression was analyzed in our cultures (48 hrs of treatment with Pom), the cell viability (% of live cells) remained high (average viability > 88% of vehicle treated cells for both 1 and 10 μM) (Supplementary Figure S2A) and there was only modest reduction in the number of viable cells over the time period of the experiment compared to the vehicle control (79% and 63% in butyrate and butyrate/Pom-treated cells, respectively) (Supplementary Figure S2B). This indicates that these effects on MHC-I were not simply due to the toxic effects of these compounds. Pom also prevented the downregulation of MHC-I induced by TPA another lytic inducer of viral replication (Supplementary Figure S1C and S1D) suggesting the effect was not simply due to blocking the butyrate signaling pathway.

**Figure 5:**
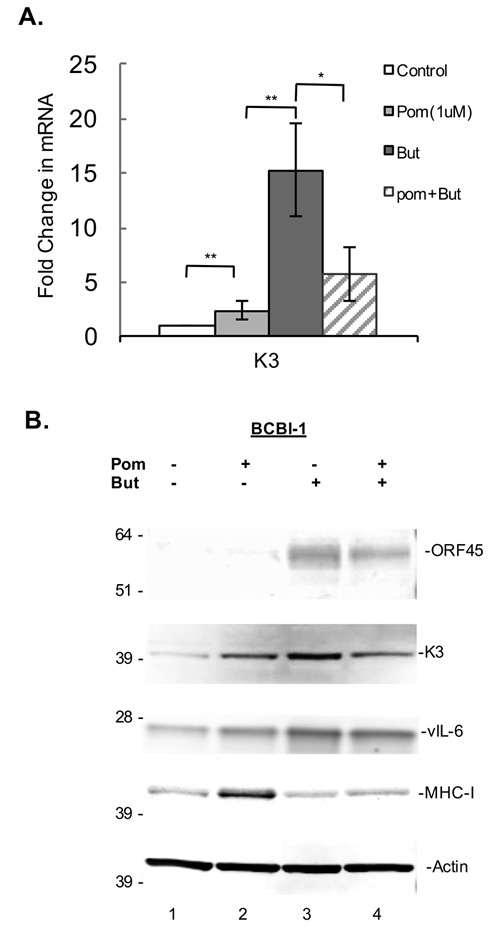
Effect of Pom on K3 mRNA gene expression and effects on protein expression in latent and lytic BCBL-1 cells BCBL-1 cells were treated with control (DMSO) or Pom (1 μM) for 24 h followed by treatment with PBS or butyrate for an additional 24 h. Total mRNA was isolated and analyzed for expression levels by real time QT-PCR and was normalized to 18S levels. **A**. Shown is the fold changes in mRNA expression for K3, ** *P* < 0.01, * *P* < 0.05, for two tailed paired Student's *t*-test. **B**. Immunoblot analysis of whole cell lysate protein (20 μg) for ORF45, K3, vIL-6, MHC-I of BCBL-1 cells treated with vehicle controls, Pom 1 μM, Butyrate (But) 0.3 mM, or Pom/But. Actin was run as a loading control.

We also investigated the effect of Pom on the cytoplasmic levels of MHC-I. Cells were treated without or with 10 μM Pom for 24 h followed by treatment with 0.3 mM butyrate for 24 h. Cytoplasmic extracts were prepared and analyzed by immunoblot. Pom alone caused a discernable increase in MHC-I levels in cytoplasmic extracts of BCBL-1 and BC-3 cells even in the absence of butyrate (Figure 2E and 2F). Quantification relative to tubulin levels revealed that butyrate treatment substantially decreased cytoplasmic levels of MHC-I and this decrease was substantially prevented by pretreatment of cells with Pom (Figure 2G). In addition, this analysis showed that cytoplasmic levels of MHC-I increased over basal levels in cells with Pom alone (Figure 2G) although this effect on the cytoplasmic levels was less noticeable at 1 μM Pom (see Figure 5B).

### Pom and Len restore ICAM-1 and B7-2 surface expression in uninduced BCBL-1 cells

**Figure 3:**
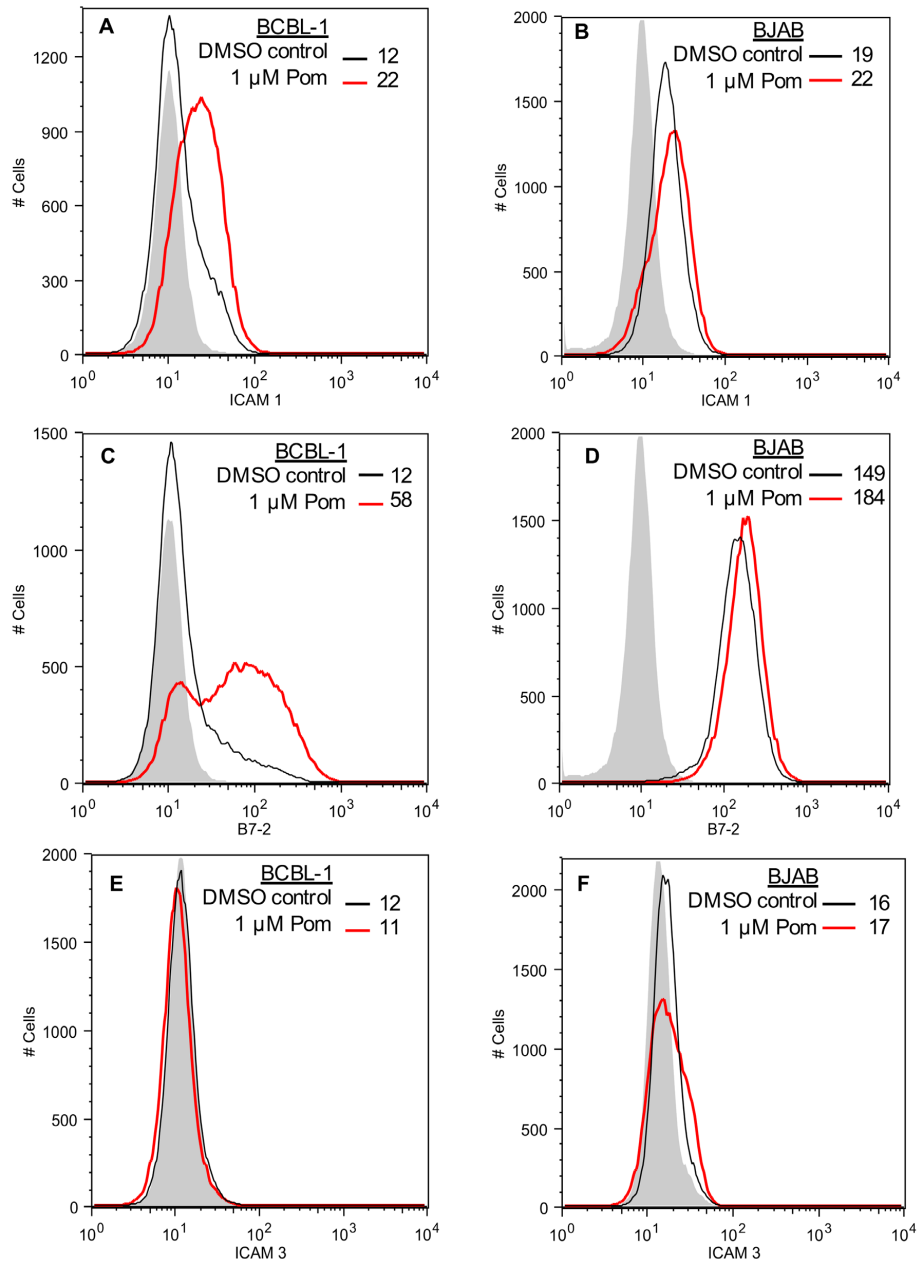
Restoration of ICAM-1 and B7-2 but not ICAM-3 expression in BCBL-1 cells treated with Pom **A**. ICAM-1 expression in BCBL-1 cells and in **B**. BJAB cells as determined by FACS after pretreatment for 48 h with DMSO vehicle control or Pom (1 μM). **C**. B7-2 expression in BCBL-1 cells and in **D**. BJAB cells as determined by FACS after pretreatment for 48 h with DMSO vehicle control or Pom (1 μM). **E**. ICAM-3 expression in BCBL-1 cells and in **F**. BJAB cells as determined by FACS after pretreatment for 48 h with DMSO vehicle control or Pom (1 μM). The data shows a representative experiment of four independent experiments for **A**. and **C**. and two independent experiments for **B**., **D**., and **F**. with similar results. The grey shading shows the isotype control.

**Figure 4:**
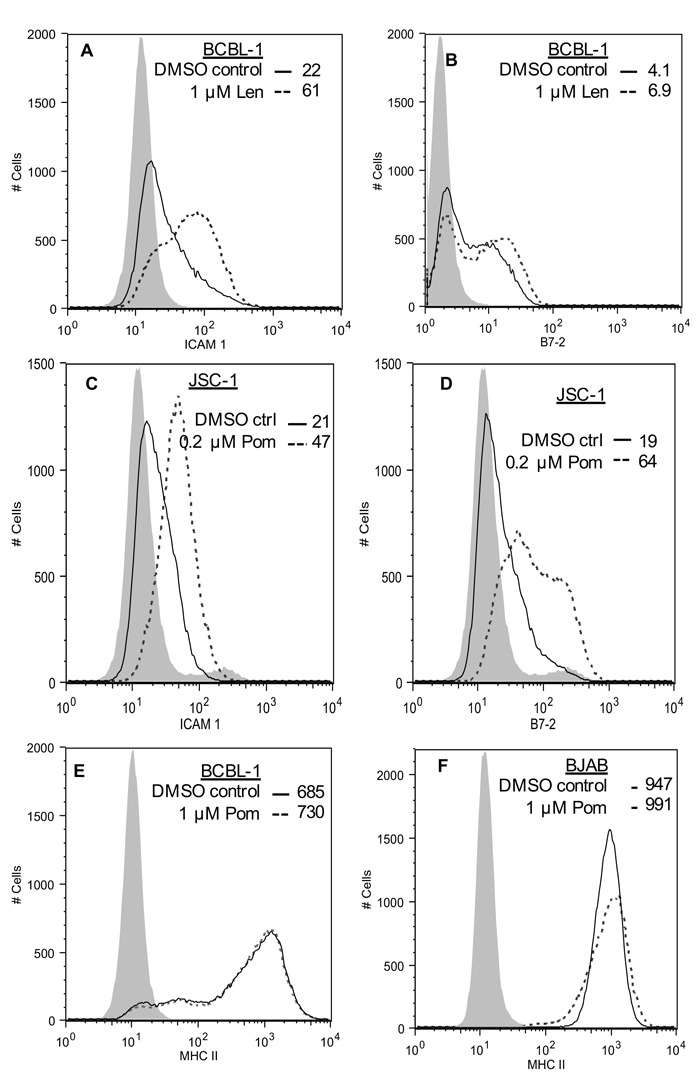
Effect of Len on ICAM-1 and B7-2 expression in BCBL-1 cells, effect of Pom on MHC-II expression in BCBL-1 and BJAB cells, and effect of Pom on ICAM-1 and B7-2 in JSC-1 cells **A**. ICAM-1 expression and **B**. B7-2 expression in BCBL-1 cells pretreated for 48 h with DMSO vehicle control or Pom (1 μM) as determined by FACS. **C**. ICAM-1 expression **D**. B7-2 expression in JSC-1 cells pretreated for 48 h with DMSO vehicle control or Pom (0.2 μM) as determined by FACS. **E**. MHC-II expression in BCBL-1 cells or **F**. BJAB cells pretreated for 48 h with DMSO vehicle control or Pom (1 μM) as determined by FACS. The data is representative of two independent experiments in A, one experiment in B-D, and two experiments in E and F. The grey shading shows the isotype control.

KSHV also down-regulates ICAM-1 and B7-2, which are ligands for NK cell-mediated cytotoxicity receptors [7, 11]. These ligands are undetectable or poorly expressed in PEL lines and latently infected cells [11, 32, 33]. The mechanism responsible for their decreased expression in latent cells is not completely clear, but K5 is expressed at low levels in latently infected cells and has been shown to down-regulate these ligands in model systems [7, 11, 29]. We investigated the effects of 1 μM Pom and Len on ICAM-1 and B7-2 expression in unstimulated PEL lines. In BCBL-1 cells, these ligands were virtually undetectable (Figure 3A and 3C), while they were readily detectable in KSHV-uninfected BJAB cells (Figure 3B and 3D). Treatment of BCBL-1 cells with Pom increased ICAM-1 to levels close to that seen in untreated BJAB (Figure 3A and 3B). B7-2 expression was also increased but remained less than that observed for untreated BJAB cells (Figure 3C and 3D). Pom had little or no effect on the levels of ICAM-1 or B7-2 in BJAB cells (Figure 3B and 3D, red line). Len (1 μM) also increased ICAM-1 and B7-2 expression in BCBL-1 cells (Figure 4A and 4B). ICAM-1 and B7-2 also increased in Pom-treated JSC-1 cells (Figure 4C and 4D). By contrast to these effects on ICAM-1 and MHC-I, expression of ICAM-3 and MHC-II, which are not targets of K3 or K5, were unaffected by Pom (Figure 3E and 4E). Thus, Len and Pom selectively restore expression of immune recognition proteins in latent and lytic PEL cells.

### Differential effects of Pom on latent and lytic KSHV gene expression

To better understand the mechanism by which Pom increases the expression of these immune surface receptors, we sought to investigate its effects on K3 and K5 gene expression in both latent and lytic induced cells. Although the viral mechanisms by which latent cells decrease ICAM-1 and B7-2 expression are not completely clear, there is evidence that K3 and K5 are the primary mediators of MHC-I down-regulation in lytic cells [7, 9, 34]. In addition, K5 is known to downregulate ICAM-1 and B7-2 in transfected cell models [11] and therefore may play a role in downregulating these ligands in latently infected cells. BCBL-1 cells were pretreated 24h with 1 μM Pom and then induced to lytic replication with 0.3 mM butyrate. The gene expression levels for K3 as well as the protein levels of K3, ORF45 (an immediate early lytic protein), vIL-6 (an early lytic protein), and MHC-I were then assessed 24h later (Figure 5A and 5B). MHC-I increased in cells treated with Pom alone and increased in cells exposed to Pom and butyrate as compared with butyrate alone (Figure 5B). Treatment of BCBL-1 cells with Pom led to a small but statistically significant increase (*P* < 0.05) in K3 gene expression over control levels (Figure 5A). This corresponded to an increase in K3 protein expression as well (Figure 5B). In cells induced with sodium butyrate, K3 expression increased substantially (Figure 5A); however, pretreatment with Pom decreased K3 mRNA and protein expression in butyrate-induced cells (Figure 5A and 5B). Thus, in butyrate-induced cells Pom decreases K3 mRNA expression and this may contribute to the Pom-induced upregulation of MHC-I. We sought to assess the changes in K5 gene expression, but it was at the limit of detection with our primers and we were not able to reliably determine changes in expression with butyrate and/or Pom. As expected, ORF45, an immediate early lytic protein, was strongly induced by butyrate and Pom prevented some of the butyrate-induced increase of this lytic gene as well (Figure 5B). However, vIL-6, another lytic gene that increased with butyrate was only weakly decreased by Pom treatment in the presence of butyrate (Figure 5B) suggesting that Pom may selectively affect certain viral lytic genes.

### Effect of Pom on K3 and K5 induced down-regulation of MHC-I in transfected BJAB cells

**Figure 6:**
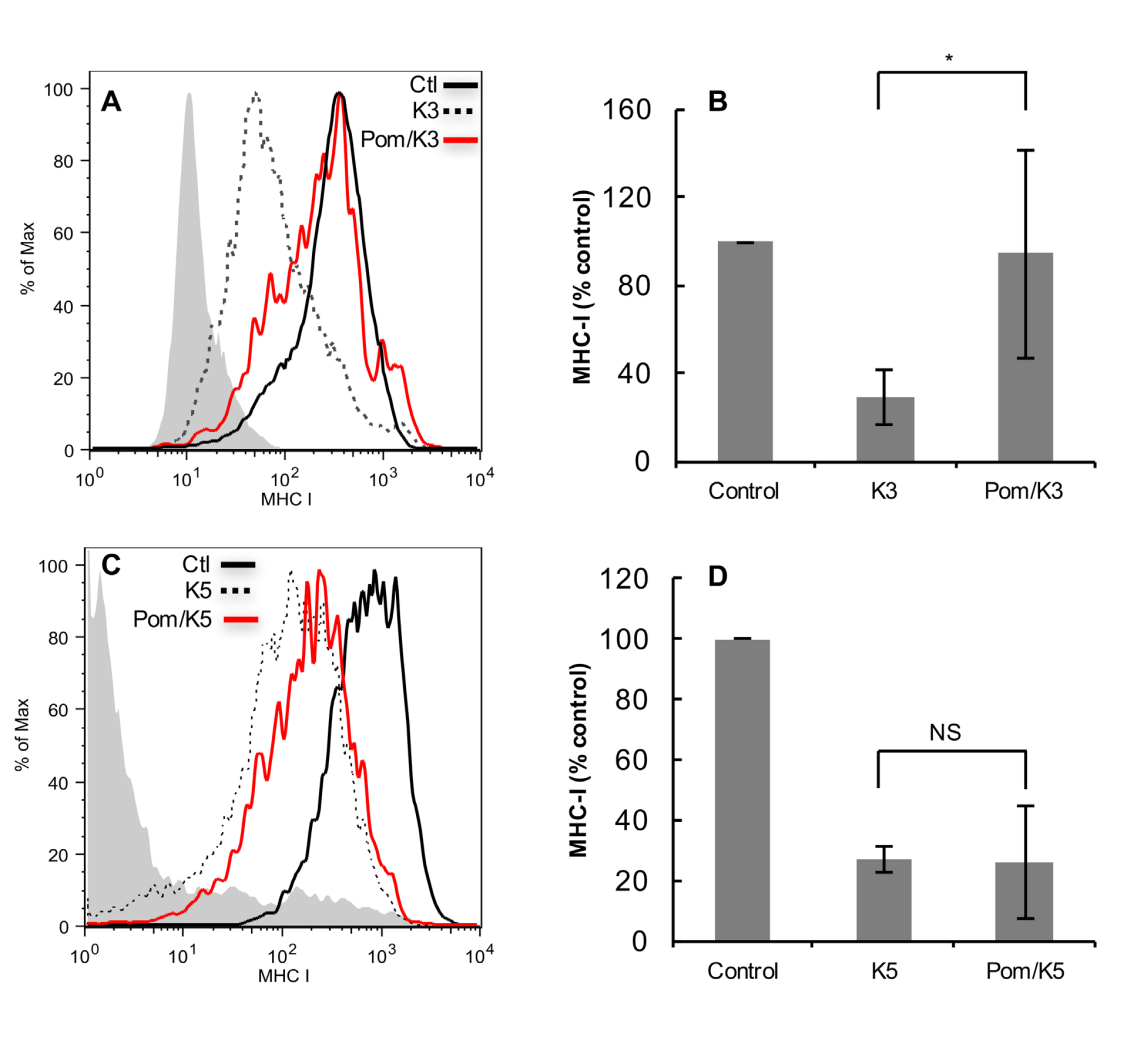
Effect of Pom on down-regulation of MHC-I in BJAB cells transfected with K3 or K5 FACS analysis showing the effect of GFP-K3 and GFP-K5 transfection on MHC-I down regulation in BJAB cells without or with pretreatment (48 hr) with Pom (10 μM). **A**. Cells were transfected with GFP plasmid control or GFP-expressing K3 and MHC-I levels were assessed 24 h after transfection on cells expressing similar levels of GFP (gated for GFP) as determined by FACS to control for protein expression levels. The grey shading shows the isotype control in A and C. Shown is a representative experiment. Median fluorescent values for DMSO control, K3, and Pom/K3 were 334, 67 and 246, respectively. **B**. Data compiled on the MHC-I expression in control, K3 and Pom/K3 treated cells as described above from 4 separate experiments. Median fluorescent values for DMSO control, K3, and Pom/K3 were 757, 126 and 195, respectively. **C**. Cells were transfected with GFP plasmid control or GFP-expressing K5 and MHC-I levels were assessed on cells expressing similar levels of GFP, shown is a representative experiment. **D**. Data compiled on the MHC-I expression in control, K5 and Pom/K5 treated cells as described above from 3 separate experiments. Values plotted are the mean of the median fluorescence values +/- standard deviation. Statistics comparing K3 or K5 with Pom-treated cells were performed using Student's two tailed paired *t*-test, *P < 0.05.

Since KSHV-encoded K3 and K5 are both expressed during lytic replication and play a major role in down-regulating MHC-I [7], we further explored the effects of Pom in K3- and/or K5-transfected BJAB cells. Previously, K3 and K5 expression in BJAB cells was shown to decrease expression of MHC-1 and several other surface immune molecules [34]. Cells were transfected with a plasmid encoding either GFP-K3 or GFP-K5. Expression of GFP-K3 or GFP-K5 in BJAB cells showed a substantial down-regulation of MHC-I expression (Figure 6A and 6C, dashed lines). Pretreatment of cells with Pom almost completely inhibited down-regulation of MHC-I by K3 (on average by 95%; *P* < 0.05) (Figure 6B). However, Pom only slightly prevented down-regulation in GFP-K5-transfected cells (Figure 6C and 6D).

### Pom prevents a decrease in HLA class I mRNA expression during lytic activation

**Figure 7:**
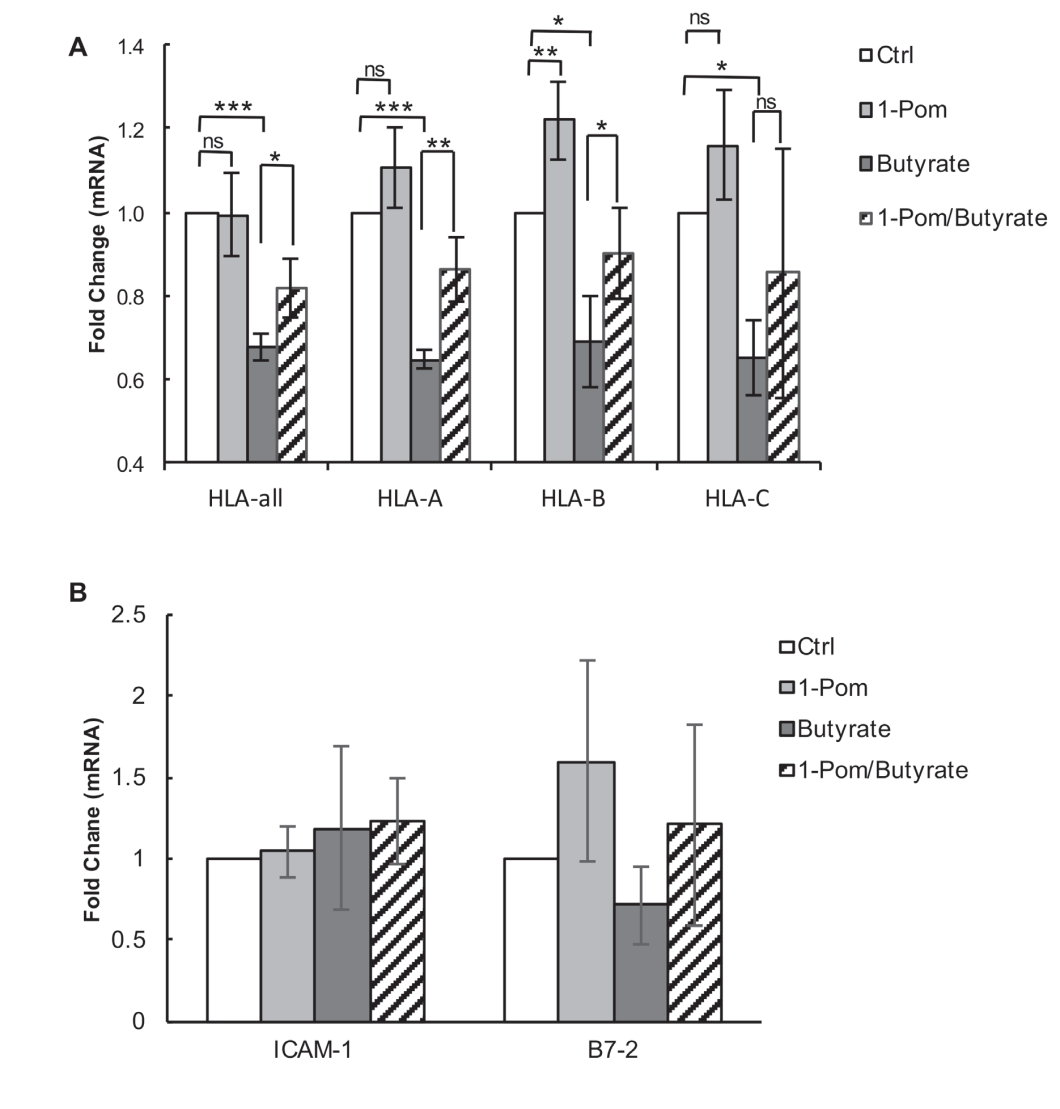
Effect of Pom on MHC-I (HLA-A, B and C), ICAM and B7-2 mRNA gene expression in BCBL-1 cells induced with butyrate BCBL-1 cells were treated with control (DMSO) or Pom (1 μM) for 24 h followed by treatment with PBS or butyrate for an additional 24 h. Total mRNA was isolated and analyzed for expression levels by real time QT-PCR and was normalized to 18S levels. Shown are the fold changes in mRNA expression for total **A**. HLA and HLA A, B and C alleles following treatment and **B**. ICAM-1 and B7-2. Values are the average +/- standard deviation of four independent experiments. *** *P* < 0.001, **P < 0.01, *P < 0.05, for two tailed paired Student's t-test. ns = not significant. Tests were not significantly different for comparisons within ICAM-1 and B7-2 samples.

Previous studies have shown that KSHV-encoded proteins can affect MHC-I expression both at the level of synthesis [13] and degradation [7, 8, 29, 32, 34]. In addition to K3 and K5, which down-regulate MHC-I by increasing degradation, vIRF-1, a lytic gene of KSHV also expressed at low levels in latency, has been shown to suppress MHC-I by decreasing expression of MHC-I mRNA [13]. We found that treatment of BCBL-1 with butyrate decreased expression of total HLA mRNA and HLA subtypes A, B and C (Figure 7A). However, when cells were pretreated with Pom for 24 h prior to butyrate, almost half of the butyrate-induced decrease in mRNA expression was prevented. This was statistically significant for the combination of HLAs (*P* < 0.05), for HLA-A (*P* < 0.01), and for HLA-B (*P* < 0.05), but not for HLA-C (Figure 7A). Pom was most effective at restoring HLA-A mRNA (Figure 7A). However, Pom alone had little effect on the basal mRNA expression levels of HLA-A and HLA-C and only modestly increased HLA-B in uninduced cells (*P* < 0.01) (Figure 7A). We explored whether restoration of HLA mRNA expression in butyrate-treated PEL cells by Pom coincided with a reduction of the lytic switch gene, RTA, and vIRF-1 mRNA expression. However, the effect of Pom on these lytic genes was not significant (Supplementary Figure S3). Pom alone did not induce RTA over control levels. Also, vFLIP, which enhances MHC-I transcription in KSHV infected cells [13], did not consistently increase in the presence of Pom without or with butyrate (Supplementary Figure S3). We also determined whether the restoration of ICAM-1 and B7-2 surface expression was a result of increased mRNA expression for these proteins. However, Pom had no significant effect on ICAM-1 expression (Figure 7B). Although there was a trend of increased B7-2 mRNA expression following Pom treatment in the absence or presence of butyrate, the changes were not significant (Figure 7B) suggesting these drugs do not act at the level of synthesis to restore these surface molecules.

### Pom treatment is associated with inhibition of cell growth and decreases in Ikaros, Aiolos and IRF4 expression, but does not induce interferon gamma (IFNγ)

**Figure 8:**
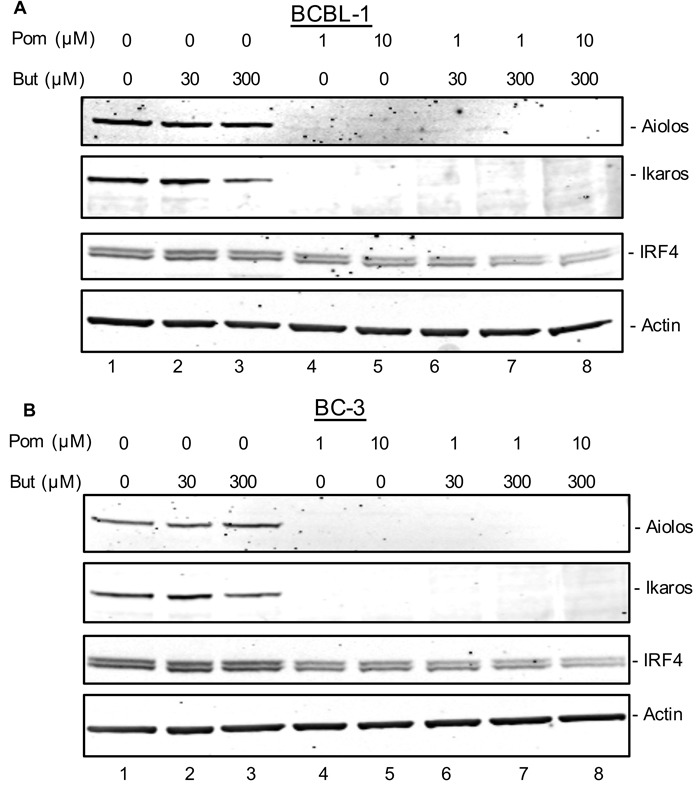
Effect of Pom on Ikaros, Aiolos, and IRF-4 expression in latent and lytic BCBL-1 and BC-3 cells **A**. BCBL-1 or **B**. BC-3 cells were treated with vehicle control (DMSO) or Pom (1 μM and 10 μM) for 24 h. Cells were then treated with butyrate (30 μM or 300 μM) to induce lytic activation or treated with PBS as a control. After 24 h, extracts were prepared and analyzed by immunoblot. **A**. Protein (20 μg) from BCBL-1 cells and **B**. BC-3 cells were analyzed by immunoblot for Ikaros, Aiolos, and IRF-4 following treatment with butyrate (30 μM and 300 μM), Pom (1 μM and 10 μM) or the combination of both butyrate and Pom. Actin was used as a loading control. Shown is a representative experiment from 2 separate experiments for each cell line.

Len and Pom alter substrate specificity for cereblon, leading to changes in expression of proteins including decreases in Ikaros, Aiolos, IRF4, and cMyc, and increases in IFNγ and interlukin-2 (IL-2) [17–21]. Some of the effects of Len and Pom on cell proteins, such as increases in IFNγ [35, 36], could also potentially be responsible for changes observed in MHC-I expression in PEL cells. To explore this, we investigated whether Pom and Len affected expression of Aiolos, Ikaros, and IRF4 in latent and lytic PEL cells. Cells were pretreated with Pom for 24 h and then induced to lytic gene expression with butyrate or treated with PBS. Nuclear extracts were analyzed for Aiolos, Ikaros, and IRF4 by immunoblot. Aiolos and Ikaros were detectable in latent and lytic BCBL-1 and BC-3 cells, although butyrate treatment somewhat reduced expression of Ikaros and Aiolos (Figure 8A and 8B, lanes 1-3. Treatment of latent and lytic BCBL-1 and BC-3 cells with Pom resulted in a dramatic loss of Ikaros and Aiolos (Figure 8A and 8B, lanes 4-8). Loss of Ikaros and Aiolos also coincided with a decrease in IRF4 expression (Figure 8A and 8B, lanes 4-8). Len, tested at 1μM and 10 μM, also substantially decreased expression of Ikaros and Aiolos in latent and lytic BCBL-1 and BC-3 cells (Supplementary Figure S4A and S4B). Previous studies have shown that these changes in Ikaros and Aiolos can coincide with increased expression of IFNγ in certain cells [35, 36]. Since IFNγ can increase MHC-I expression in response to intracellular pathogens [37] we assessed IFNγ expression after treatment with Pom. No IFNγ (limit of detection 16 pg/ml) could be detected in BCBL-1 supernatants or BC-3 PEL cells treated with Pom at 0.1, 1.0 or 10 μM for 48 h. This finding is consistent with what others have reported [24]. Thus, there was no evidence to indicate that induction of IFNγ causes the Pom-mediated prevention of down-regulation of the immune surface expression proteins MHC-I, ICAM-1 and B7-2.

## DISCUSSION

In this report, we demonstrate that immunomodulatory agents Pom and Len prevent down-regulation of MHC-I surface expression in PEL lines undergoing KSHV lytic activation. These drugs also restore ICAM-1 and B7-2 expression in the latently infected PEL lines BCBL-1 and JSC-1. Through a variety of mechanisms, KSHV encoded proteins function to decrease expression of these important immune molecules. Down-regulation of these components is an important viral immune evasion strategy that can render virus-infected cells and tumors invisible to cytotoxic T cells and NK cells, respectively. The ability of Pom and Len to reverse these processes provides a strong rationale for their use in PEL and other KSHV-associated tumors.

Surface expression of MHC-I and other surface proteins in infected cells represents a balance between production and cellular or virus-mediated destruction. We found that Pom increased MHC-I mRNA expression during lytic activation, and this is likely one mechanism that contributes to the prevention of MHC-I downregulation during the lytic cycle. However, since the effects on MHC-I mRNA expression may not be responsible for all the surface expression changes in MHC-I, we explored other possible mechanisms. Pom was found to decrease K3 mRNA expression in lytic cells, thus providing an additional mechanism by which these drugs can enhance MHC-I expression in lytic cells. By contrast, restoration of ICAM-1 and B7-2 by Pom did not appear to occur through increased mRNA expression and it remains possible that these drugs may indirectly inhibit the function of the low level of K5 present in latent cells. It is interesting to note that K5 expression has been observed in the absence of ORF50 (RTA) expression [38, 39]. K3 and K5 are ubiquitin ligases, and while cereblon is one of many proteins that provide substrate specificity to RING E3 ubiquitin ligases, it is conceivable that Len and Pom also have a direct effect on K3 and/or K5, or that these viral proteins also interact with cereblon. The ability of Pom to reverse MHC-I downregulation by K3 transfection, but not K5 transfection, provides some suggestive evidence that it might have a more direct effect on K3-mediated MHC-downregulation. However, it is possible that the downregulation mediated by K5 transfection of BJAB cells simply overwhelmed the ability of Pom to restore MHC-I. Additional research will be needed to explore whether Len or Pom interfere directly with the activities of K3 or K5.

Another question is how these drugs blunt the decrease in MHC-I mRNA expression otherwise observed during lytic activation. An important downstream effect of the binding of these drugs to cereblon is a decrease in the activity of Ikaros and Aiolos; this in turn leads to suppression of IRF4 and cMyc, cell toxicity, and increases in IFNγ and IL-2 [17–21]. Since IFNγ stimulates production of MHC-I [35], it was possible that increases in IFNγ could be responsible for changes observed in MHC-I expression in PEL cells. However, consistent with previous reports [24], IFN-γ was undetectable in PEL lines, suggesting this is not a contributing mechanism. However, degradation of the transcriptional repressors Ikaros and Aiolos may increase MHC-I transcription through undetermined mechanisms. Interestingly, Hagner et al. reported that CC-122, a related cereblon-binding drug, increases transcription of IFN-stimulated genes independent of increases in IFN-α, IFN-β, or IFN-γ [40], so a similar mechanism may contribute to these effects. Thus, multiple mechanisms may exist by which Len and Pom prevent down-regulation of MHC1, and restore ICAM-1, and B7-2.

Consistent with previous reports [24], we found that Tha, Len, and Pom had some anti-proliferative effects on PEL cells at the doses used here, which were associated with decreases in IRF4. However, the effects on MHC-1 that we observed were not likely to be due to toxicity, as cell viability remained almost unchanged in the period in which we assessed surface expression. Also, from the perspective of anti-cancer therapy, an increase in MHC-I would be beneficial in a setting of tumor cell toxicity. We also considered that the drugs might be causing cell death through induction of KSHV lytic replication. A knockdown in IRF4 can lead to lytic reactivation of KSHV in PEL cells [41], and Tha, Len, and Pom have all been reported to cause lytic activation of Epstein Barr virus [41]. However, we and others [24] only observed a modest increase in lytic activation of KSHV in Pom-treated PEL cells and there was no significant effect on the lytic switch gene RTA. Also, we consistently observed inhibition of lytic protein production in cells induced to lytic activation with butyrate.

The results presented here demonstrate that in addition to their broad co-stimulation of effector T cells, Len and Pom also restore expression of important immune receptors on KSHV-infected tumor cells. Because viruses introduce foreign antigens, virus-induced tumors are potentially highly susceptible to immune-based therapies. However, a major challenge to this approach is that nearly all viruses have evolved strategies to render infected cells invisible to the immune system, particularly a down-regulation of MHC-I and other surface receptors such as ICAM-1 or B7-2. By preventing down-regulation of MHC-I, Pom and Len could render KSHV-associated tumors susceptible to cytotoxic T cells, and by up-regulating ICAM-1 and B7-2 could also increase the cells susceptibility to NK cells, which would otherwise destroy the cells if MHC-I alleles are not expressed. In this regard, previous studies have demonstrated that de novo expression of B7-2 and ICAM-1 restores NK cell-mediated killing of K5-expressing cells [11]. Further studies will be required to understand the degree to which restoring these surface molecules render these cells susceptible to cytotoxic T cells and to NK cells.

These effects of Len and Pom may be particularly useful from a clinical perspective, as it is focused on the virus-infected tumor cells rather than non-specifically activating the immune system with the associated risk of autoimmune toxicities. Also, because these drugs are affecting MHC-1 on the tumor cells, it may be worth exploring their use with an anti-PD-1, anti-PD-L1, or anti-CTLA-4 checkpoint inhibitors, which enhance the activity of effector T cells [42, 43]. A similar strategy has recently been proposed with MAP-kinase inhibitors, which also have been reported to enhance expression of MHC-I in certain tumor cell lines [44].

Based on their anti-angiogenic and broad immunomodulatory activities Tha, Len, and Pom have been studied in patients with KS and all have shown activity [25, 26, 45–48] and Len has been reported to have activity in a patient with PEL [49]. In particular, Pom was shown to induce clinical responses in most KS patients with acceptable toxicity [26]. It is quite possible that inhibition of KSHV-induced MHC-I, ICAM-1, and B7-2 down-regulation contributes to this anti-KS activity. In summary, we demonstrate that Len and Pom prevent down-regulation of MHC-I and restore ICAM1 and B7-2 in KSHV infected cells, a novel discovery that can lead the way in therapeutic strategies to thwart virus-mediated immune evasion in PEL and other KSHV-induced disorders. Also, given that the mechanism for prevention of immune surface marker downregulation may be at least in part independent of specific effects on KSHV-encoded genes, these drugs may have utility in other viral-induced tumors and diseases. Additional studies will be needed to asses this possibility.

## MATERIALS AND METHODS

### Cell culture

BC-3 (ATCC, Manassas, VA) and BCBL-1 cells (National Institutes of Health AIDS Research and Reagent Program, Rockville, MD) and two uninfected Burkitt lymphoma B-cell lines, CA-46 and BJAB (ATCC, Manassas, VA) and JSC-1 cells (gift from Richard Ambinder, John Hopkins University, Baltimore, MD) were maintained as described previously [50]. Stocks (20 mM in DMSO) of Tha, Len, and Pom (Celgene Corp., Summit, NJ) were stored frozen.

### Flow cytometry analysis and FACS antibodies

Cells were analyzed with a FACScalibur™ Flow Cytometry system (BD Biosciences, San Jose, CA). Antibodies used were: FITC-anti-HLA clone w6/32 (recognizes HLA-A, B and C) (Sigma-Aldrich, St. Louis, MO), PE-anti-HLAdr MHC-II (Biolegend, San Diego, CA), FITC-ICAM-1, FITC-ICAM-3 (Santa Cruz, Santa Cruuz, CA), FITC-B7-2 and its FITC-IgG2a isotype control (Sigma-Aldrich). FACS was performed on BJAB cells transfected with GFP-K3 (gift from Dr. Jae Jung) [7] or GFP-K5 encoding plasmids. To produce GFP-K5, K5 was amplified by PCR from KSHV-infected BCBL-1 viral DNA using primers K5F: CTA AGA TCT ATG GCG TCT AAG GAC GTA and K5R: CGC GAA TTC ACC GTT GTT TTT TGG ATG containing BglII and EcoRI restriction sites at the 5’ and 3’ ends, respectively. Products were digested with BglII and EcoRI, cloned into green fluorescent protein (GFP)-tagged pEGFP-C1 (Clontech, CA) and designated GFP-K5. BJAB cells, 300,000 cells/ml, were treated with drug or DMSO. After 48 hours, cells were electroporated with GFP control vector for K3 (pTracer-Ve), GFP K3 vector plasmid (pTracer-K3), GFP vector plasmid for K5 (pEGFP-Ctl), or a K5 expression vector pEGFP-K5. Cells (500,000) were washed, suspended in 4D-Nucleofector buffer SF with supplement with 0.25 μg DNA was transferred to 16-well 20 μL Nucleocuvette™ strips. Electroporation was performed in a Lonza 4D-Nucleofector™ X unit. After electroporation (24 h), GFP and MHC-I were assessed by flow cytometry. Surface expression markers were determined after gating the live cell population. MHC-I levels in GFP transfected cells were assessed using PE-conjugated antibodies to HLA Class I clone w6/32 (Sigma-Aldrich). Control and drug treated cells were analyzed by gating cells expressing similar GFP levels.

### Immunoblotting

Whole cell lysates were prepared with m-per (Pierce, Rockland, IL). In some cases, nuclear and cytoplasmic extracts were prepared using NE-PER Nuclear Extraction Reagent kit (Pierce) with protease inhibitors (Halt Protease Inhibitors Cocktail, Pierce). Protein concentrations were determined using the BCA assay (Pierce). Samples were subjected to LDS-PAGE (4 to 12 % NuPAGE Tris-Bis) (Invitrogen, Carlsbad, CA) and transferred to a nitrocellulose membrane with iBlot (Life Technologies Grand Island, NY). Membranes were blocked overnight with 5% w/v nonfat dry milk in 1X TBST or Odyssey blocking buffer (Licor, Lincoln, NE). Primary antibodies used were: mouse anti-β-actin, mouse anti-β-tubulin, mouse anti-CRBN, or mouse anti-Ikaros (IKZF-1) (Sigma-Aldrich); mouse anti-IRF-4, rabbit anti-p21, mouse anti-MHC-I, mouse anti-Aiolos (IKZF-3) (Santa Cruz Biotechnology, Dallas, TX); mouse anti-open reading frame 45 (ORF45) (Abcam, Cambridge, UK), rabbit monoclonal antibody to vIL-6 [51], mouse anti-K3 (gift from Dr. Jae Jung, USC); or mouse anti-K5 (gift from Dr. Ueda, Osaka University). Secondary antibodies were conjugated to alkaline phosphatase (Promega, Madison. WI) or conjugated to green or red fluorescent dyes for use with LI-COR system.

### Real-time quantitative reverse transcription PCR on KSHV infected Cells

Cells were plated (3 × 10^5^ cells per mL) and treated with 1 μM Pom. After treatment (24h) cells were treated with 0.3 mM butyrate (Sigma-Aldrich) (24 h) and RNA extracted using mirVana™ kit (ThermoFisher Scientific, Waltham MA). cDNA synthesis was performed using High-Capacity cDNA Reverse Transcription kit (ThermoFisher Scientific) on a T100 Thermal Cycler (Bio-Rad). q-PCR reaction setup included enzyme activation at 95°C for 10 min and 40 cycles of amplification at 95°C for 15 sec and 60°C for 1 min followed by melting curve analysis. Expression was normalized to 18S endogenous control RNA and quantification of relative mRNA expression was performed using ∆∆Cт method. The following primers (5’ to 3’) were used in this study for q-PCR:

18S: GCCCGAAGCGTTTACTTTGA and TCCATTATTCCTAGCTGCGGTATC, K3: CCCTGTGCATCCACAGGG and GAGCCAGGTGCTTAAACAAC, HLA-all: GCGGCTACTACAACCAGAGC and GATGTAATCCTTGCCGTCGT, HLA-A: AAGGCCCACTCACAGACTGA and ACTTGCGCTTGGTGATCTGA, HLA-B: CTACCCTGCGGAGATCA and ACAGCCAGGCCAGCAACA, HLA-C: CACACCTCTCCTTTGTGACTTCAA and CCACCTCCTCACATTATGCTAACA, RTA: GTCATGTCACCCTTGCGATC and ACGCTTCTTTGAGCTCCTCT, vIRF1: GTCTCTGCGCCATTCAAAAC and CCGGACACGACAACTAAGAA, vFLIP: CGTCTACGTGGAGAACAGTGAGCT and CTGGGCACGGATGACAGGGAAGTG. Primers for ICAM-1 and B7-2 were from Biorad (ICAM-1: 10025636, qHsaCED0004281) and (B7-2: 10025636, qHsaCED0043530 (Hercules, CA).

### Statistics

Experiments performed at least 3 times show the mean and standard deviation. Statistical analysis was done using the Students two tailed paired *T*-test on experiments that were performed four times or more.

## SUPPLEMENTARY MATERIALS FIGURES



## Abbreviations

DMSO: dimethyl sulfoxide
FACS: fluorescent activated cell sorter
Tha: Thalidomide
Len: Lenalidomide
Pom: Pomalidomide
KSHV: Kaposi's sarcoma associated herpesvirus
MHC-I: major histocompatibility class I
ICAM-1: Intercellular Adhesion Molecule 1
PEL: primary effusion lymphoma
KS: Kaposi's sarcoma
NK: natural killer
vFLIP: viral FLICE inhibitory protein
CUL4: cullin-4
IRF-4: interferon regulatory factor 4
BRD4: replication and transcription activator
RTA: bromo domain 4
LANA: latency associated nuclear antigen
GFP: green fluorescent protein
HLA: human leukocyte antigen
IFN-γ: interferon gamma
IL-2: interlukin 2
RING: really interesting new gene

## References

[1] Soulier J, Grollet L, Oksenhendler E, Cacoub P, Cazals-Hatem D, Babinet P, d’Agay MF, Clauvel JP, Raphael M, Degos L, Sigaux F (1995). Kaposi's sarcoma-associated herpesvirus-like DNA sequences in multicentric Castleman's disease. Blood.

[2] Cesarman E, Chang Y, Moore PS, Said JW, Knowles DM (1995). Kaposi's sarcoma-associated herpesvirus-like DNA sequences in AIDS-related body-cavity-based lymphomas. N Engl J Med.

[3] Chang Y, Cesarman E, Pessin MS, Lee F, Culpepper J, Knowles DM, Moore PS (1994). Identification of herpesvirus-like DNA sequences in AIDS-associated Kaposi's sarcoma. Science.

[4] Polizzotto MN, Uldrick TS, Wyvill KM, Aleman K, Marshall V, Wang V, Whitby D, Pittaluga S, Jaffe ES, Millo C, Tosato G, Little RF, Steinberg SM (2016). Clinical Features and Outcomes of Patients With Symptomatic Kaposi Sarcoma Herpesvirus (KSHV)-associated Inflammation: Prospective Characterization of KSHV Inflammatory Cytokine Syndrome (KICS). Clin Infect Dis.

[5] Kwun HJ, da Silva SR, Qin H, Ferris RL, Tan R, Chang Y, Moore PS (2011). The central repeat domain 1 of Kaposi's sarcoma-associated herpesvirus (KSHV) latency associated-nuclear antigen 1 (LANA1) prevents cis MHC class I peptide presentation. Virology.

[6] Haque M, Chen J, Ueda K, Mori Y, Nakano K, Hirata Y, Kanamori S, Uchiyama Y, Inagi R, Okuno T, Yamanishi K (2000). Identification and analysis of the K5 gene of Kaposi's sarcoma-associated herpesvirus. J Virol.

[7] Ishido S, Wang C, Lee BS, Cohen GB, Jung JU (2000). Downregulation of major histocompatibility complex class I molecules by Kaposi's sarcoma-associated herpesvirus K3 and K5 proteins. J Virol.

[8] Lorenzo ME, Jung JU, Ploegh HL (2002). Kaposi's sarcoma-associated herpesvirus K3 utilizes the ubiquitin-proteasome system in routing class major histocompatibility complexes to late endocytic compartments. J Virol.

[9] Boname JM, Lehner PJ (2011). What has the study of the K3 and K5 viral ubiquitin E3 ligases taught us about ubiquitin-mediated receptor regulation?. Viruses.

[10] Krishnan HH, Naranatt PP, Smith MS, Zeng L, Bloomer C, Chandran B (2004). Concurrent expression of latent and a limited number of lytic genes with immune modulation and antiapoptotic function by Kaposi's sarcoma-associated herpesvirus early during infection of primary endothelial and fibroblast cells and subsequent decline of lytic gene expression. J Virol.

[11] Ishido S, Choi JK, Lee BS, Wang C, DeMaria M, Johnson RP, Cohen GB, Jung JU (2000). Inhibition of natural killer cell-mediated cytotoxicity by Kaposi's sarcoma-associated herpesvirus K5 protein. Immunity.

[12] Sun Z, Jha HC, Pei YG, Robertson ES (2016). The MHC II HLA-DRalpha is Downregulated by KSHV encoded lytic transactivator RTA and MARCH8. J Virol.

[13] Lagos D, Trotter MW, Vart RJ, Wang HW, Matthews NC, Hansen A, Flore O, Gotch F, Boshoff C (2007). Kaposi sarcoma herpesvirus-encoded vFLIP and vIRF1 regulate antigen presentation in lymphatic endothelial cells. Blood.

[14] Qian Z, Zhang L, Cai Z, Sun L, Wang H, Yi Q, Wang M (2011). Lenalidomide synergizes with dexamethasone to induce growth arrest and apoptosis of mantle cell lymphoma cells in vitro and in vivo. Leuk Res.

[15] Zhang L, Qian Z, Cai Z, Sun L, Wang H, Bartlett JB, Yi Q, Wang M (2009). Synergistic antitumor effects of lenalidomide and rituximab on mantle cell lymphoma in vitro and in vivo. Am J Hematol.

[16] Zhu YX, Kortuem KM, Stewart AK (2013). Molecular mechanism of action of immune-modulatory drugs thalidomide, lenalidomide and pomalidomide in multiple myeloma. Leuk Lymphoma.

[17] Lopez-Girona A, Mendy D, Ito T, Miller K, Gandhi AK, Kang J, Karasawa S, Carmel G, Jackson P, Abbasian M, Mahmoudi A, Cathers B, Rychak E (2012). Cereblon is a direct protein target for immunomodulatory and antiproliferative activities of lenalidomide and pomalidomide. Leukemia.

[18] Zhu YX, Braggio E, Shi CX, Bruins LA, Schmidt JE, Van Wier S, Chang XB, Bjorklund CC, Fonseca R, Bergsagel PL, Orlowski RZ, Stewart AK (2011). Cereblon expression is required for the antimyeloma activity of lenalidomide and pomalidomide. Blood.

[19] Ito T, Ando H, Suzuki T, Ogura T, Hotta K, Imamura Y, Yamaguchi Y, Handa H (2010). Identification of a primary target of thalidomide teratogenicity. Science.

[20] Gandhi AK, Kang J, Havens CG, Conklin T, Ning Y, Wu L, Ito T, Ando H, Waldman MF, Thakurta A, Klippel A, Handa H, Daniel TO (2014). Immunomodulatory agents lenalidomide and pomalidomide co-stimulate T cells by inducing degradation of T cell repressors Ikaros and Aiolos via modulation of the E3 ubiquitin ligase complex CRL4(CRBN). Br J Haematol.

[21] Bjorklund CC, Lu L, Kang J, Hagner PR, Havens CG, Amatangelo M, Wang M, Ren Y, Couto S, Breider M, Ning Y, Gandhi AK, Daniel TO (2015). Rate of CRL4(CRBN) substrate Ikaros and Aiolos degradation underlies differential activity of lenalidomide and pomalidomide in multiple myeloma cells by regulation of c-Myc and IRF4. Blood Cancer J.

[22] Yang Y, Shaffer AL, Emre NC, Ceribelli M, Zhang M, Wright G, Xiao W, Powell J, Platig J, Kohlhammer H, Young RM, Zhao H, Xu W (2012). Exploiting synthetic lethality for the therapy of ABC diffuse large B cell lymphoma. Cancer Cell.

[23] Lopez-Girona A, Heintel D, Zhang LH, Mendy D, Gaidarova S, Brady H, Bartlett JB, Schafer PH, Schreder M, Bolomsky A, Hilgarth B, Zojer N, Gisslinger H (2011). Lenalidomide downregulates the cell survival factor, interferon regulatory factor-4, providing a potential mechanistic link for predicting response. Br J Haematol.

[24] Gopalakrishnan R, Matta H, Tolani B, Triche T, Chaudhary PM (2016). Immunomodulatory drugs target IKZF1-IRF4-MYC axis in primary effusion lymphoma in a cereblon-dependent manner and display synergistic cytotoxicity with BRD4 inhibitors. Oncogene.

[25] Little RF, Wyvill KM, Pluda JM, Welles L, Marshall V, Figg WD, Newcomb FM, Tosato G, Feigal E, Steinberg SM, Whitby D, Goedert JJ, Yarchoan R (2000). Activity of thalidomide in AIDS-related Kaposi's sarcoma. J Clin Oncol.

[26] Polizzotto MN, Uldrick TS, Wyvill KM, Aleman K, Peer CJ, Bevans M, Sereti I, Maldarelli F, Whitby D, Marshall V, Goncalves PH, Khetani V, Figg WD (2016). Pomalidomide for Symptomatic Kaposi's Sarcoma in People With and Without HIV Infection: A Phase I/II Study. J Clin Oncol.

[27] Cavert W, Notermans DW, Staskus K, Wietgrefe SW, Zupancic M, Gebhard K, Henry K, Zhang ZQ, Mills R, McDade H, Schuwirth CM, Goudsmit J, Danner SA (1997). Kinetics of response in lymphoid tissues to antiretroviral therapy of HIV-1 infection [see comments] [published erratum appears in. Science.

[28] Means RE, Ishido S, Alvarez X, Jung JU (2002). Multiple endocytic trafficking pathways of MHC class I molecules induced by a Herpesvirus protein. Embo J.

[29] Haque M, Ueda K, Nakano K, Hirata Y, Parravicini C, Corbellino M, Yamanishi K (2001). Major histocompatibility complex class I molecules are down-regulated at the cell surface by the K5 protein encoded by Kaposi's sarcoma-associated herpesvirus/human herpesvirus-8. J Gen Virol.

[30] Chen N, Lau H, Kong L, Kumar G, Zeldis JB, Knight R, Laskin OL (2007). Pharmacokinetics of lenalidomide in subjects with various degrees of renal impairment and in subjects on hemodialysis. J Clin Pharmacol.

[31] Kasserra C, Assaf M, Hoffmann M, Li Y, Liu L, Wang X, Kumar G, Palmisano M (2015). Pomalidomide: evaluation of cytochrome P450 and transporter-mediated drug-drug interaction potential in vitro and in healthy subjects. J Clin Pharmacol.

[32] Means RE, Lang SM, Jung JU (2007). The Kaposi's sarcoma-associated herpesvirus K5 E3 ubiquitin ligase modulates targets by multiple molecular mechanisms. J Virol.

[33] Gregory SM, Wang L, West JA, Dittmer DP, Damania B (2012). Latent Kaposi's sarcoma-associated herpesvirus infection of monocytes downregulates expression of adaptive immune response costimulatory receptors and proinflammatory cytokines. J Virol.

[34] Li Q, Means R, Lang S, Jung JU (2007). Downregulation of gamma interferon receptor 1 by Kaposi's sarcoma-associated herpesvirus K3 and K5. J Virol.

[35] Davies FE, Raje N, Hideshima T, Lentzsch S, Young G, Tai YT, Lin B, Podar K, Gupta D, Chauhan D, Treon SP, Richardson PG, Schlossman RL (2001). Thalidomide and immunomodulatory derivatives augment natural killer cell cytotoxicity in multiple myeloma. Blood.

[36] Corral LG, Haslett PA, Muller GW, Chen R, Wong LM, Ocampo CJ, Patterson RT, Stirling DI, Kaplan G (1999). Differential cytokine modulation and T cell activation by two distinct classes of thalidomide analogues that are potent inhibitors of TNF-alpha. J Immunol.

[37] Schroder K, Hertzog PJ, Ravasi T, Hume DA (2004). Interferon-gamma: an overview of signals, mechanisms and functions. J Leukoc Biol.

[38] Okuno T, Jiang YB, Ueda K, Nishimura K, Tamura T, Yamanishi K (2002). Activation of human herpesvirus 8 open reading frame K5 independent of ORF50 expression. Virus Res.

[39] Paulose-Murphy M, Ha NK, Xiang C, Chen Y, Gillim L, Yarchoan R, Meltzer P, Bittner M, Trent J, Zeichner S (2001). Transcription program of human herpesvirus 8 (kaposi's sarcoma-associated herpesvirus). J Virol.

[40] Hagner PR, Man HW, Fontanillo C, Wang M, Couto S, Breider M, Bjorklund C, Havens CG, Lu G, Rychak E, Raymon H, Narla RK, Barnes L (2015). CC-122, a pleiotropic pathway modifier, mimics an interferon response and has antitumor activity in DLBCL. Blood.

[41] Forero A, Moore PS, Sarkar SN (2013). Role of IRF4 in IFN-stimulated gene induction and maintenance of Kaposi sarcoma-associated herpesvirus latency in primary effusion lymphoma cells. J Immunol.

[42] Mahoney KM, Freeman GJ, McDermott DF (2015). The Next Immune-Checkpoint Inhibitors: PD-1/PD-L1 Blockade in Melanoma. Clin Ther.

[43] Ott PA, Hodi FS, Robert C (2013). CTLA-4 and PD-1/PD-L1 blockade: new immunotherapeutic modalities with durable clinical benefit in melanoma patients. Clin Cancer Res.

[44] Brea EJ, Oh CY, Manchado E, Budhu S, Gejman RS, Mo G, Mondello P, Han JE, Jarvis CA, Ulmert D, Xiang Q, Chang AY, Garippa RJ (2016). Kinase Regulation of Human MHC Class I Molecule Expression on Cancer Cells. Cancer Immunol Res.

[45] Rubegni P, Sbano P, De Aloe G, Flori ML, Fimiani M (2007). Thalidomide in the treatment of Kaposi's sarcoma. Dermatology.

[46] Ben M’barek L, Fardet L, Mebazaa A, Thervet E, Biet I, Kerob D, Morel P, Lebbe C (2007). A retrospective analysis of thalidomide therapy in non-HIV-related Kaposi's sarcoma. Dermatology.

[47] Soler RA, Howard M, Brink NS, Gibb D, Tedder RS, Nadal D (1996). Regression of AIDS-related Kaposi's sarcoma during therapy with thalidomide. Clin Infect Dis.

[48] Martinez V, Tateo M, Castilla MA, Melica G, Kirstetter M, Boue F (2011). Lenalidomide in treating AIDS-related Kaposi's sarcoma. AIDS.

[49] Antar A, El Hajj H, Jabbour M, Khalifeh I, El-Merhi F, Mahfouz R, Bazarbachi A (2014). Primary effusion lymphoma in an elderly patient effectively treated by lenalidomide: case report and review of literature. Blood Cancer J.

[50] Davis DA, Singer KE, Reynolds IP, Haque M, Yarchoan R (2007). Hypoxia enhances the phosphorylation and cytotoxicity of ganciclovir and zidovudine in Kaposi's sarcoma-associated herpesvirus infected cells. Cancer Res.

[51] Hu D, Wang V, Yang M, Abdullah S, Davis DA, Uldrick TS, Polizzotto MN, Veeranna RP, Pittaluga S, Tosato G, Yarchoan R (2016). Induction of Kaposi's Sarcoma-Associated Herpesvirus-Encoded Viral Interleukin-6 by X-Box Binding Protein 1. J Virol.

