# Supplementary material for: Restoration of immune surface molecules in Kaposi sarcoma-associated herpes virus infected cells by lenalidomide and pomalidomide
Source: Oncotarget. 2017 May 17;8(31):50342–58. doi: 10.18632/oncotarget.17960 (PMC5584136; doi:10.18632/oncotarget.17960)
Supplement: Supplementary file 1 [file oncotarget-08-50342-s001.pdf]

Restoration of immune surface molecules in Kaposi sarcoma-associated herpes virus infected cells by lenalidomide and pomalidomide

Supplementary Material

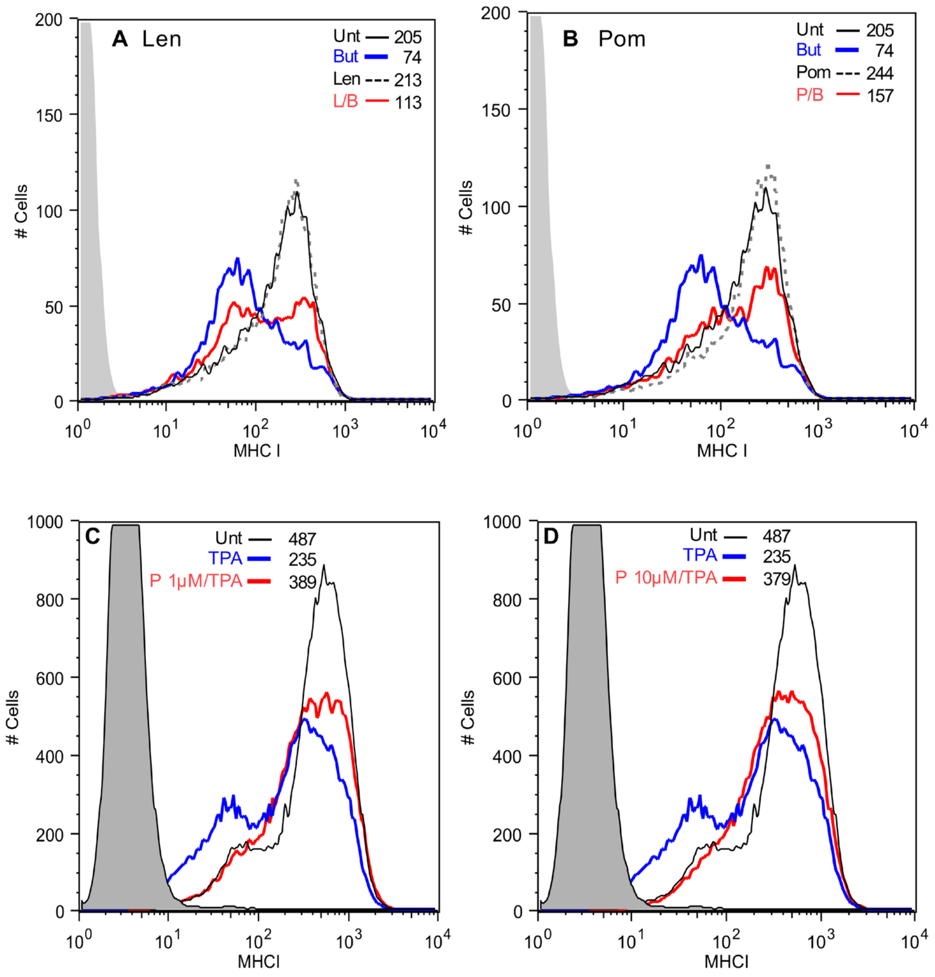

Supplementary Figure S1: Effect of Len and Pom on the lytic-induced down-regulation of MHC-I in KSHV infected cells at clinically achievable levels and effect of Pom on TPA induced downregulation of MHC-I. Cells (300,000 cells/ml in 10 ml) were pretreated for 24 h with Len or Pom or DMSO vehicle control. Cells were then treated with sodium butyrate (0.3 mM) or 7.5 nM TPA for another 24 h and then analyzed by flow cytometry for surface protein expression. Cells were washed with cold FACS buffer (10% FBS, 0.1% sodium azide in PBS) and incubated with conjugated monoclonal antibodies (2 µL) for 30 min at 4°C. After washing twice, MHC-I expression was determined by FACS. (A) Cells pretreated for 24 h with DMSO vehicle control (Unt) or Len (L) 2 µM (Cmax) followed by treatment with PBS or 0.3 mM butyrate (B). Cells pretreated for 24 h with DMSO vehicle control (Unt) or Pom (P) 0.3 µM (Cmax) followed by treatment with PBS or 0.3 mM butyrate. The grey shaded area in each panel represents the isotype control. Butyrate treated cells are in the blue tracing and cells pretreated with the drugs (L/B, and P/B) are in red tracing. (C) Cells pretreated for 24 h with DMSO vehicle control (Unt) or Pom (P) at 1 µM or (D) 10 µM followed by treatment with 7.5 nM TPA. TPA-treated cells are in the blue tracing and cells pre-treated with are in red tracing. The median fluorescent values are indicated within each Figure.

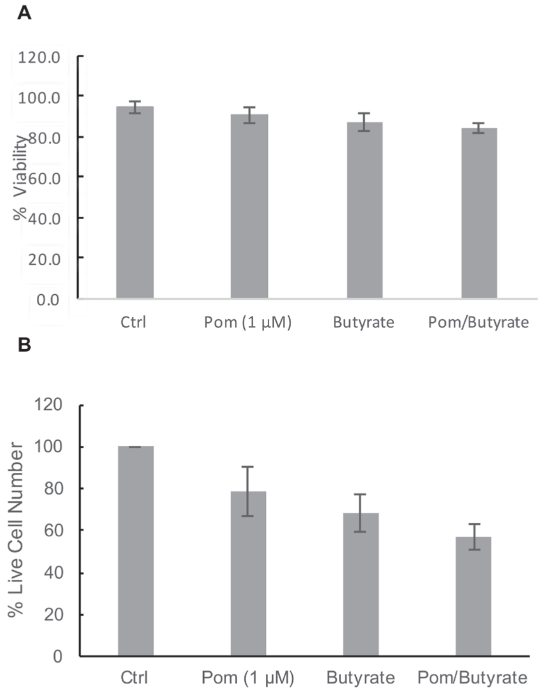

Supplementary Figure S2: Effect Pom, butyrate and Pom/butyrate on cell viability and live cell number. Cells (300,000 cells/ml in 10 ml) were pretreated for 24 h with Pom (1 µM) or DMSO vehicle control. Where indicated, cells were then treated with sodium butyrate (0.3 mM) for another 24 h and then cell viability and live cell numbers were determined by trypan blue staining at the 48 hr timepoint. (A) Cell viability (live cell number over total cells) presented as percent control cell viability and (B) Live cell number presented as a percent of control live cell number. The data presented represents the average and standard deviation from 5 separate experiments.

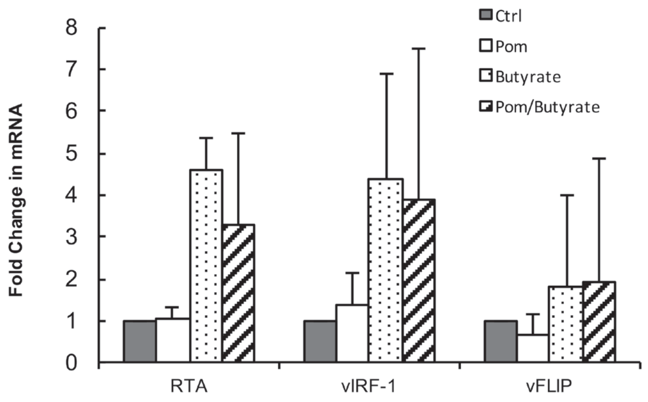

Supplementary Figure S3: Effect of Pom on RTA, vIRF-1 and vFLiP mRNA expression in BCBL-1 cells induced with butyrate. BCBL-1 cells were treated with control (DMSO) or Pom for 24 h followed by treatment with PBS or butyrate for an additional 24 h Total mRNA was isolated and analyzed for expression levels by real time QT-PCR and normalized to 18S levels. Values are the average +/- standard deviation of four independent experiments. Shown are fold change in RTA, vIRF-1 and vFLIP mRNA levels following treatments. The primers used were (5' to 3'):

18S: GCCCGAAGCGTTTACTTTGA and TCCATTATTCCTAGCTGCGGTATC
RTA: GTCATGTCACCCTTGCGATC and ACGCTTCTTTGAGCTCCTCT
vIRF1: GTCTCTGCGCCATTCAAAAC and CCGGACACGACAACTAAGAA
vFLIP: CGTCTACGTGGAGAACAGTGAGCT and CTGGGCACGGATGACAGGGAAGTG.

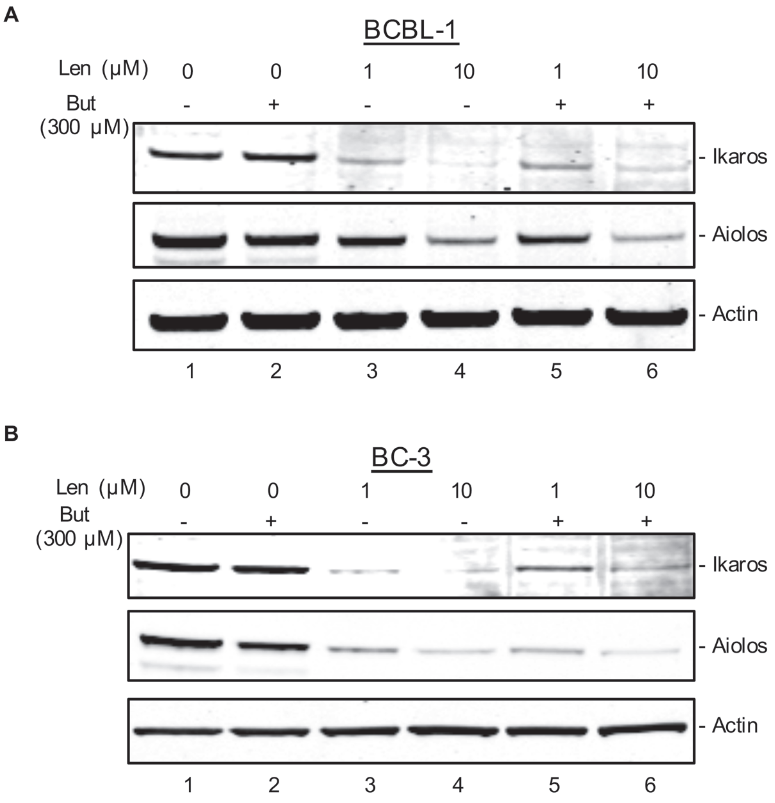

Supplementary Figure S4: Effect of Len on Ikaros and Aiolos expression in latent and lytic BCBL-1 and BC-3 cells. (A) BCBL-1 or (B) BC-3 cells were treated with vehicle control (DMSO) or Len (1 μM and 10 μM) for 24 h Cells were then treated with 300 μM butyrate to induce lytic activation or treated with PBS as a control. After 24 h extracts were prepared and analyzed by immunoblot for Ikaros and Aiolos. Actin was used as a loading control. Shown is a representative experiment from 2 separate experiments for each cell line.
